# The British Journals, &c.: Pathology, Practical Medicine, and Therapeutics

**Published:** 1842-07

**Authors:** 


### PATHOLOGY, PRACTICAL MEDICINE, AND THERAPEUTICS.

On the Globules in Health and Disease. The first solid compound of vital affinity beyond the boundary of health, is coagulable lymph, which follows in straight succession on the effusion of serosity; and in ordinary circumstances pus is the second. It is between the one and other of the two, that tubercle seems deservedly to occupy a place. The morbid preparation is, to appearance, nearly the same for all of them; and the secretion of each respectively may go on simply from the mass yet in circulation, or be attended with ultimate congestion of blood of a permanent kind. But beyond this they differ widely. The globule of pus is an orbicular membrane, full of a more fluid matter; organizable lymph puts on the cellular and fibrous arrangement; and tubercle, with all its points of resemblance to the former in the early periods, and to coagulated albumen, and particles of fibrine in the later periods, gradually changes from a fluid to a solid friable mass, only to soften again with the solution of the texture it occupies, resolving into globular bodies of considerable consistence, and sufficiently characteristic. When secreted, so like the product of plasticity, and fully formed, resembling much that of suppuration—it yet attains to neither. It goes progressively through the course of generating, maturing, and softening tubercle; the ultimate symptom of scrofula.

In addition to some of the appearances of tubercle formerly described by the author, the following are given: In the maturer form of yellow matter, it presents the most satisfactory appearances. When compressed between the object-glasses with a drop of water, it bruises with more or less facility, assuming a milky look, and a part of it composed of globular fragments mixes with the fluid. The entire mass appears granular in the microscope, and of a light sienna colour. The borders are irregular, jagged, and broken, and many projecting fragments, about to separate themselves, are apparently attached to it by softer and more elastic matter, resembling in some measure a connecting tissue. This is not unlike what we have seen uniting the particles of fibrinous substance, which joins the fibres of muscle previously boiled. At an earlier stage, this softer material of union is less remarkable, because of the greater homogenity of the morbid matter. When, however, it approaches towards softening, the connecting tissue grows more elastic and apparent, but to disappear ultimately in leaving innumerable globular bodies.

Besides the mass of globular bodies, fibres of a particular sort are found in tuberculized lungs. They are most arranged at angles to one another, and for the most part united in bundles of about half a dozen threads. In sound parenchyma, these fibres are lax, huddled together, and the threads indistinct: in the diseased lung, the mesh of tissues being full of tubercle, they are separated by the morbid matter. The author seems disposed to believe that the interrupted respiration of commencing phthisis may be owing to some irregular and irritable contraction of those fibres.

Most probably expectorated tubercle is very often the portions of tuberculized lung, which are less advanced in softening, but separated away by dissolution of the mass surrounding them. Their form is often lenticular, but not regularly so; they are mostly broader than thick, having thereby, at times, a wafer-looking shape, as if they were portions of the tubercle lining cavities, and we have found bodies exactly similar in extensive excavations of the lungs. They are opaque-white, yellowish-white, and grayish-white, according as it may be. Some of them are firmer in comparison to others; but generally they are of soft consistence, and easily compressed by the object-glasses of the microscope. They are evidently softer when expectorated than on their separation from the mass of lung. Again, they have more consistence than particles of ripe tubercle about to loosen altogether into globules. This is probably owing to their primitive quality on being cast off from the mass of disease. We have seen the firmest tubercles soften by prolonged maceration in water during summer; and this accords with our opinion that it is for the most part imperfectly matured tubercle, which gains the exterior in morsels mixed in sputa.

The following ingredients have been found, by the author, in the sputa of phthisical patients; shot-like granules, as large as swan-shot, sometimes of the size of a minute pea: epithelium in layers; a globular mass, composed of an aggregation of eight or a dozen saccular-looking globules, of the volume of colchicum seeds, which cannot be traced to any morbid process in particular; various crystals, one sort having the form of a wafer the size of a sixpence, with a polished scaly appearance, and a variegated yellowish and slightly brown colour; another resembling three pins placed at right angles to each other, with their points joining; again, a larger crystal of parallel prisms; vegetable matter, which most generally occurs in the form of long stalks, of the thickness of blades of grass to that of the stem of dandelion; sometimes they seem to have joints, and to terminate in an asparagus-looking head.—Dr. Watts, *Dublin Journal of Medical Science,* No. lxi. *March,* 1842.

---

Diagnosis and Treatment of certain Cardiac Affections. If we examine the heart and pericardium, when removed from the subject, seeing the former collapsed, and loosely surrounded by the latter, we cannot understand how various morbid sounds can be produced, by the motion of the one within the other. But such is not the condition of those parts in the living body; the pericardium is there firmly fixed at its apex and base, and it is tense and stretched, like the parchment of a drum; and if in this bag we have an enlarged heart, moving slowly backwards and forwards, and if the internal surface of that bag have been previously the seat of morbid deposits or effusions, punctiform or ridge-like, such conditions of the cardiac sac must cause very varying sounds emanating from the same. Pericarditic sounds may be as *loud and as prolonged as valvular sounds,* a fact hitherto scarcely sufficiently dwelt on by pathologists. Pericarditic sounds, like valvular, may be accompanied by *frémissement;* and consequently, in endeavouring to make the diagnosis between the two sets of sounds, we must keep in view the fact, that pericarditic sounds appear, to the careful ear, to issue from a more superficial source, to be more extensively diffused, and to be almost equally audible in regions of the chest very distant from each other; as for instance, under both clavicles. Pericarditic sounds, too, undergo much quicker alteration in character than valvular, and seem to be conducted by the solid parietes of the chest, while valvular sounds are chiefly propagated by the contents and parietes of the great vessels.

The author gives a case, which he considers to be "unique" in this respect, that the rheumatic inflammation seized the pericardium *before* the joints. This fact proves that physicians have been too apt to attribute pericarditis, carditis, or endocarditis, to metastasis, a doctrine applicable to some cases, but by no means to all. Dr. G. conceives that rheumatic fever, an inflammation *sui generis, may exist without inflammation of the joints, or arthritis; as, on the contrary, arthritis may exist without rheumatic fever.* This view he founds on his observation of the fact, that he has noticed, in the same patients, a fever having all the characters of rheumatic, but which in some cases was accompanied with, and in other cases existed without, inflammation of a single joint.

In some cases of pericarditis the heart's action becomes increased in strength for many hours before any physical signs of pericarditis can be detected, and before any pain is felt in the region of the heart. In such cases, when the usually acknowledged symptoms of pericarditis are added to this already existing augmented action of the heart, the latter goes on increasing, and finally becomes excessively violent, and does not begin to decrease notably for several days after the peculiar symptoms of pericarditis have disappeared. This course may, perhaps, be explained by supposing that the muscular substance of the heart became inflamed before the pericarditis came on, and continued to be so after the pericarditis subsided.

The author gives it as his opinion that the functional derangements produced by disease of any particular part of the heart, are seldom sufficiently characteristic to enable us to make out whether the disease be situated in the auriculo-ventricular or semilunar valves, and owns that it has frequently occurred to him that all the symptoms supposed to be indicative of disease of the right side of the heart, have been occasioned by disease of the left side, and *vice versa.*

As to the motions of the heart, their derangement scarcely ever, according to Dr. Graves, indicates the seat of disease with any precision.—Dr. Graves, *Dublin Journal of Medical Science*, No. lxii. *May,* 1842.

---

Cancer of the Lungs and Mediastinum. In this paper a number of interesting cases, collected and original, are given. The author sums up as follows:

1. That the facility of diagnosis mainly depends on the anatomical disposition of the disease.

2. That we may divide the cases with a view to diagnosis into those in which isolated tubercles exist, with the intervening tissues healthy; those in which simple degeneration occurs without ulceration, and with ulceration; and those in which a tumour of the mediastinum exists, causing compression.

3. That the diagnosis in the first case is difficult, from our being seldom able to avail ourselves of the signs of irritation and ulceration, so important in ordinary tubercles, and the fact of the equable distribution of the disease preventing comparison.

4. That in some cases of isolated cancerous masses, the diagnosis may be founded on the same general principles as that of acute phthisis.

5. That in simple cancerous degenerations of the lung, the principal physical signs are the gradual diminution of the vesicular murmur, without râle; its ultimate extinction; and the signs of perfect solidification.

6. That the evidences of perfect solidification are better found in this disease than in any other pulmonary affection.

7. That this form of the disease may exist, simply, or in combination with empyema, and may be secondary to cancerous tumours of the mediastinum.

8. That the sides may be symmetrical in this affection, and that either dilatation or contraction of the side may occur.

9. That the mediastinum may be displaced, even though the side be contracted.

10. That under these circumstances we may have the signs of perfect solidification, accompanied by imperfect pectoriloquism, and increased vibration to the hand.

11. That the mediastinum may be displaced and the liver depressed without protrusion of the intercostal spaces.

12. That the heart may be compressed and dislocated in this form of disease.—*Hughes, Syms, Houston.*

13. That the flattening of the upper part of the chest may occur from degeneration of the upper lobe.—*Hughes.*

14. That the absence of signs of ulceration is very characteristic of this disease.

15. That we have observed these signs in but a single case, and that the phenomena, though they might be produced by other diseases causing the same physical conditions of the lung, have never before been met with.

16. That cancerous tumours of the mediastinum generally coexist with either degeneration of the lung, or isolated tubercles in its substance.

17. That they may be solid or fluid.

18. That they may coexist with cancerous infiltration of the lung, or the deposit of cancer in the bronchial tubes.

19. That they are to be recognized more by the signs of the tumour, than by those of disease of the lung.

20. That dysphagia, tracheal stridor, feebleness of one pulse, difference of respiratory murmur from pressure on the bronchial tube, displacement of the diaphragm, and dilatation of the heart, may occur in this form of the disease.

21. That a cancerous tumour may exhibit pulsation with or without bellows murmur, but that pulsation is not always attendant on it

22. That though the previous existence of external cancer may assist in diagnosis, yet that the disease may be all through internal, or the visceral precede the external cancer.

23. That the feebleness of pulsation connected with the extent of dulness may assist in distinguishing the disease from aneurism.

24. That in the advanced periods, as in aneurism, gangrene of a portion of the lung may supervene.

25 That the following symptoms are important as indicative of this disease: pain of a continued kind; a varicose state of the veins in the neck, thorax, and abdomen; œdema of one extremity; rapid formation of external tumours of a cancerous character; expectoration similar in appearance to currant jelly; resistance of symptoms to ordinary treatment.

26. That though none of the physical signs of this disease are, separately considered, peculiar to it, yet *that their combinations and modes of succession* are not seen in any other affection of the lung.—Dr. Stokes, *Dublin Journal of Medical Science*, No. lxii. *May*, 1842.

---

Pathology of Typhus. Rokitansky first boldly announced the doctrine that the typhous product is as peculiar, and stands in the same relation to the disease producing it, as the matter of scirrhus or tubercle does to the morbid affections producing these formations; and that, in the case of typhus, this cause is a *dyscrasia*, expressing itself by a tendency to the deposition, in certain localities, more especially in the ileum, of a substance as peculiar in its nature as the matter of scirrhus or tubercle. The authors, then, in drawing this alleged analogy between tuberculosis and typhus, point out the peculiar mode and character of the tubercular deposit, and affirm that precisely similar to this, is what Rokitansky teaches to be the typhous process. In one important particular they seem to differ. Tuberculosis is a chronic, typhus a rapid disease; in the former, moreover, there are always distinct local symptoms, and seldom general fever; the contrary, in the latter. But if we could conceive a variety of tuberculosis, equally rapid in its course as typhus, presenting no obvious local symptom, and attended with violent fever, the resemblance between this disease and typhus would be obvious. Now it so happens there *is* such a disease, which has been adverted to by some of the most recent writers under the name of acute tuberculosis. One case has been witnessed by the authors, which was characterized by great prostration, dry tongue, burning skin, quick pulse, stupor amounting almost to coma, without any indication of the peculiar disease being discoverable either by general symptoms or the stethoscope. This disease generally occurs in the course of chronic phthisis, but sometimes as an independent affection. The appearances, on dissection, are an almost universal dissemination of very small tubercles over the surface of the lungs, liver, kidneys, and generally also over the arachnoid membrane.

There are two theories as to the cause of typhus: the one of which is, that the disease is an essential one, without any local lesion to account for the symptoms, and that any appearances of local affection which present themselves are merely incidental, and neither cause nor characterize the disease. The other view is, that they are symptomatic of inflammation of the intestines, or of some other organ. Against the first view, it is urged that in some forms of the disease a lesion of the intestines, quite peculiar, is generally present; and although it is doubtful whether the disease can exist without the lesion, it is certain that the lesion never exists without the disease. This proves the lesion to be, if not the pathological cause, yet at least the anatomical symptom of the disease. On the other hand, it is urged that these local changes are not always present; that the severity of the disease does not correspond to their greater or less development, &c. Now Rokitanski explains these seeming anomalies, by holding that as the scrofulous diathesis may be present without the formation of tubercle, so typhous dyscrasia may exist with very little, or even altogether without typhous matter. The local lesion may be small or null, but the dyscrasia which the lesion indicates is great. Continental typhus is generally characterized by deposits, usually in the ileum; British typhus is more usually without local symptoms, and the authors cannot see how it is possible to separate them into distinct diseases, without the arbitrary division of two groups of morbid phenomena, much more closely resembling each other than is ordinarily seen in cases of any epidemic, as for example, scarlatina.—Drs. Drysdale and Russell, *London and Edinburgh Monthly Journal of Medical Science*, No. iv. *April*, 1842.

---

Antiquarian Notices of Leprosy. In this, the concluding paper on the subject, we are informed that station or rank in life was not a guarantee against the disease. It is suspected, if not certain, that the royal families both of England and Scotland had members afflicted with it. There is reason to believe that Henry IV. of England was a victim, and there is little doubt that King Robert Bruce, of Scotland, died of it, after having been for many years under its influence. Baldwin IV., king of Jerusalem, was, owing to the malady, obliged to quit the throne in his twenty-third, and perished of it in his twenty-fifth year. The male sex appears to have been the one more peculiarly subject to the disease. The affection appears also to have been clearly hereditary. As to the external exciting causes of it, these seem to have been very obscure. The Saxon habit of bathing appears to have been little imitated by the Norman conquerors; yet filth, poverty, poor diet, and wretchedness of all sorts, do not sufficiently explain the occurrence of the disease; since there are at present in Europe, and even in our own country, spots where all these conditions subsist in full force, without leprosy manifesting itself in consequence. Nor can it be explained from any varieties of temperature, climate, situation, soil, &c.; since it has been found in Sumatra, under the equator; in Iceland, within the arctic circle; in the arid plains of Arabia, and in the wet and malarious districts of Batavia and Surinam; in the interior of Africa, Hindostan, Asia Minor, and Asiatic Russia; on the sea-coast, as at Carthagena; and on the table-land of Mexico, thousands of feet above the sea-level.

It seems to be doubted, by most modern pathologists, that tubercular leprosy, as it at present exists, is of a contagious nature. When the disease is imported in the person of an infected individual, from a district where it is endemic to one where it is unknown, the malady seems to have no tendency to spread. In the Edinburgh Hospital, in 1590, two of the lepers' wives lived uninfected with their husbands, and some of the English leper hospitals served as retreats at the same time for the merely poor and for the leprous. Lepers were, in former times, regarded as defunct persons, by the civil law. Even the church took the same view of them, since the ceremonial of the burial of the dead was performed over the patient on the day on which he was consigned to the lazar-house, and separated from his healthy fellow-creatures.—Dr. J. Y. Simpson, *Edinburgh Medical and Surgical Journal*, No. cli. *April* 1, 1842.

**Valvular Diseases of the Heart.** The valvular diseases may be arranged as follows:

1. Mitral valve contracted, but capable of closing.
2. " " incapable of closing.
3. Aortic valves contracted, capable of closing.
4. " " incapable of closing.
5. Combination of 2d and 4th cases.
6. " 1st and 3d.
7. " 1st and 4th.
8. " 2d and 3d.

In all these the *rhythm* of the systole and diastole of the cavities remains as in the healthy state; the flow of blood and the sounds being shortened or prolonged in consequence of the valvular disease.

The following table shows the mode in which these sounds are produced, and the modifications they undergo in each case:

| | 1 | 2 | 3 | 4 | 5 | 6 | 7 | 8 |
|---|---|---|---|---|---|---|---|---|
| | Ventricular systole. | | | | Ventricular diastole. | | Rest. | Auricular systole. |
| Healthy state. | First sound. | | | | Second sound. | Silence. | | |
| 1 | First sound. | Silence. | | | Second sound. | Murmur. | | |
| 2 | Murmur of regurgitation. | | | | Second sound. | Murmur. | | |
| 3 | First sound, masked by murmur. | | | | Second sound. | Silence. | | |
| 4 | First sound, masked by murmur. | | | | Murmur of regurgitation. | | Silence. | |
| 5 | Double murmur. | | | | | | | Single murmur. |
| 6 | First sound, masked by murmur. | | Murmur. | | Second sound. | Murmur. | | |
| 7 | First sound, masked by murmur. | | Murmur. | | Double murmur. | | Single murmur. | |
| 8 | Double murmur. | | | | Second sound. | Single murmur. | | |

If the time of one series of the heart's actions be divided into eight parts, the ventricular systole will occupy four, during which, in the healthy state, the first sound will be audible; the ventricular diastole two, the second sound occurring at its commencement. During the systole the blood flows into the aorta, and the mitral valve closes. During the diastole, the aortic valves having shut, the blood flows into the ventricle from the auricle. The action then ceases for one eighth of the whole time, and the remaining one eighth is occupied by the silent contraction of the auricles, the blood quietly flowing into the ventricles, and thus stimulating them to contract again. It is stated by the Committee of the British Association that the systole of the auricles is accompanied by a sound; but if present, this is so slight, that in practice it may be disregarded. In the natural state, then, we have a silence of one fourth intervening *between the second and first sounds,* or before the latter.—Dr. Anderson, *Edin. Med. and Surg. Journ* No. cl.

---

Case of Hysterical Affection of the Eyes, with obstinate Closure of the Lids. About ten years ago a lady, now twenty-seven, active and healthy, was attacked, the morning following a party, with intolerance of light, pain and watering of the eyes, and then complete closure of the lids, but without spasmodic action or distortion of any kind. She was unable to open her eyelids herself, and *no force which could be employed sufficed to do so,* until she was bled to ℥viij, when they opened spontaneously; but in forty-eight hours they closed anew, to be again opened by the same means. During two years and a half the attacks recurred at irregular intervals, especially in the right eye, which never remained for more than a week unaffected; and during this period, besides venesection and arteriotomy, acupuncture of the lids, electricity, and the moxa to the vertex, were employed with success. When the eyelids were soon opened, the conjunctiva appeared natural; but when the opening was delayed, this membrane appeared flocculent and granular, and discharged a wheyish, purulent fluid. Almost every medicine in use, alteratives, tonics, antispasmodics, narcotics, have been tried, along with sea-bathing and voyages and travelling; and almost all the eminent medical men in Dublin, and several in London, have been consulted without the slightest benefit. It should have been stated that vision was perfect from the first, and still is so. The lady's circumstances are now less comfortable than formerly, and her habits more sedentary. Yet her health is good, though acupuncture does not now, as formerly, relieve. The attacks are, however, more rare.—Dr. Peebles, *Dublin Med. Press,* No. clxiv., *Feb.* 23, 1842.

---

Case of Hydrencephalocele. The patient was a boy of eleven days, and had, on each side of the upper part of the nose, a tumour; that on the right side of the size of a small plum, that on the left of a small almond. The tumours had a glossy appearance and a spongy feel, and could easily be reduced on pressure. When the finger was pressed upon them, there was an indistinct feeling as if the contents of the tumours retired into some cavity. The tumours were discovered the day after birth, at which time they were the size of a pea.

It was resolved to puncture one of these swellings, which was done accordingly. A puncture was first made with a sewing-needle, but not more than a drop of fluid escaping, the aperture was enlarged with a lancet, and a drachm of limpid fluid was discharged slowly, partly by means of pressure. When pressure was made over the tumour of the left side, the fluid flowed more freely from the other, indicating a communication betwixt them. When the child cried, the serum flowed in greater quantity, showing the tumours to communicate with the brain and to be influenced by the turgescence of the vessels of that gland. The operation was performed on June 18th, and, on the morning of the 26th, the child, after a slight convulsive fit, expired.

The dura-mater and pia-mater extended into the tumours, through openings situated below the nasal processes of the frontal bone. The anterior cornua of the lateral ventricles extended into and probably communicated with the cysts; at any rate, the cerebral substance was distinctly seen in them. The openings between the dura-mater lining the skull, and that portion of it extending into the cysts, were about the size of a crow-quill.

The author points out the possibility of such a case being mistaken for aneurism, by anastomosis, or for nævus, and in the present case, the real nature of the case was first suspected from the tumour being viewed by transmitted light, and seen to be translucent. The author would regard chronic hydrocephalus, hydrencephalocele, and spina bifida, as only three varieties of the same morbid condition; the only difference being one of extent between the two first, and of locality between them and the last one He is averse to operation in all such cases, and regrets having operated in the present. In his experience, the children operated on for hydrocephalus, or spina bifida, though healthy at the time of operation, and although every precaution was taken, have derived no benefit; febrile disturbance first, then convulsions, and finally death, have invariably ensued. He would, therefore, leave the cure to nature, the more especially as there are as many instances of natural as of artificial cure.—Wm. Lyon, Esq., *London and Edinburgh Journal of Medical Science*, No. v. *May*, 1842.

---

Violent Hysteria in a Man. This was a well-marked case, characterized by the most convulsive laughter, crying, &c. His strength was such that it required seven or eight men to hold him. There was great heat over the parietal bones, over which cold water was freely applied; and the heat on this region of the skull was generated so fast, that the cold water evaporated as if thrown on a hot substance, and rose in vapour. The man's age was twenty-six; he was small, weak, and effeminate, of an excitable temperament. It is not stated, as it ought to have been, whether he be married or not.—Alfred Smee, Esq., f.r.s., *Medical Gazette*, No. xxvii., *March* 25, 1842.

[Another and still more remarkable case is given in the 33d No. of the same Journal (May 6, 1842), by Mr. Stanger, of Nottingham. In this case the hysteric convulsions were controlled, and a cure effected, by the threat of a red-hot poker being applied to a particular part of the back. The patient was a robust-looking man, in his eighteenth year.]

---

Diabetes Mellitus: Cure (?). The patient was a man of fifty, and had been ill for a year. Had voided, during the twenty-four hours preceding the time when Mr. B. saw him, 108 ounces of straw-coloured urine of the smell and taste of honey, and of the specific gravity 1·0493. He had had cardiac palpitations during many years. Opium, quinine, and the tincture of the sesqui-chloride of iron were administered in conjunction and in a fluid form. This was on the 24th of March; and on the 10th of April a cure was effected, the urine being reduced to 42 ounces, and having lost its saccharine odour and admixture.—Mr. Joseph Bell, *Medical Gazette*, No. xxxii., *April* 29, 1842.

[If this was a case of genuine diabetes mellitus, we strongly suspect that Mr. B. will have occasion, at no distant date, to report another result.]

---

Case of Vegetable Organisms of an Undescribed form, ejected from the Stomach. A patient, nineteen years of age, had laboured for four months under stomach complaint, and every morning, ejected from his stomach, without any effort of vomiting, a quantity of fluid, varying in volume from two thirds to a whole wash-hand basinful.

On examining the fluid ejected, which smelt like fermenting wort, Mr. Goodsir expected, if he found any vegetable form at all, to see some of the globular or moniliform algæ, which it now appears pretty certain are concomitants of some of the fermentations. What was then his astonishment to find, in the first drop examined, not the vegetables he was led to expect, but numerous individuals of a form, with which the zoologist is familiar! namely an organism, which, whether animal or vegetable, was closely allied to certain genera of baccilariæ, and much more closely to the genus gonium among the volvocinæ. The author, after making the statement in the preceding sentence, and which seems to imply some doubt as to whether the organisms were animal or vegetable, speaks in the succeeding page, of "at once recognizing them as belonging to the *vegetable* kingdom." Before giving an account of the organisms, it may here be mentioned, that the formation of the morbid product was greatly controlled, and nearly put a stop to, by the use of creosote.

The following description is drawn up from examination of the ejected fluid for a period of nearly two months: In every instance the organisms presented themselves in the form of square or slightly oblong plates. The thickness of an individual was about one eighth of the length of one of its sides. Under a moderate power the sides and angles appeared straight and well defined; but under deeper glasses, the angles were rounded, and the sides sinuous; appearances which resulted from the uncompressed forms of the component cells in their particular directions.

The flat surfaces were divided into four secondary squares by two rectilinear transparent spaces, which, passing from side to side, intersected one another in the centre like two cross garden walks. Each of the four secondary squares was again divided by similarly arranged, but more feebly developed spaces, into the four ternary squares. The sixteen ternary squares thus constituted, when examined with deeper powers, were seen to consist each of four cells, which were not separated by transparent spaces, but simply by dissepiments formed by the conjunction of the walls of contiguous cells. These sixty-four cells, of which the organism consisted, did not present in perfect individuals distinct nuclei; although in certain instances appearances presented themselves, having relation to the reproduction of the organism, and falling to be described in another part of the paper. The individual organisms were transparent and slightly yellow or brown. When carefully examined under favorable circumstances the cell-walls appeared rigid, and could be perceived passing from one flat surface to the other as dissepiments. These dissepiments, as well as the transparent spaces, were from compression of contiguity rectilinear, and all the angles right angles; but the bounding cells bulged somewhat irregularly on the edges of the organism, by reason of the freedom from pressure. These circumstances gave the whole organism the appearance of a wool-pack, or of a soft bundle bound with cord, crossing it four times at right angles, and at equal distances. From these very striking peculiarities of form, I propose for it the generic term Sarcina. Perfect individual Sarcinæ, of the species now under consideration, vary from 800 to 1000 of an inch linear, along each of their sides.

Mr. Goodsir requested Dr. Wilson to subject the ejected liquid to chemical analysis; and it was found that, differently from any other liquid obtained from the human stomach, it contained at one and the same time, lactic and acetic acid. One of these acids, then was abnormal; but which? It is probable that both acids are developed during healthy digestion. Lactic acid, free or combined, abounds in the body; but acetic acid is a much rarer constituent of animal fluids, and no doubt, lactic acid has often been mistaken for it. The quantity of acetic acid found in this case was enormous.—Mr. Goodsir, *Edin. Med. and Sur. Journ.* No. 151.

---

Spontaneous Perforation of the Stomach. A woman, twenty-six years of age, unmarried and hitherto healthy, was seized with an acute pain at the epigastrium, while putting on her stays. Dr. Thompson found her labouring under excruciating pain, which she described as existing at a point about an inch and a half to the left of the xiphoid cartilage. Leeches, cupping-glasses, camomile, and poppy stupes, bran poultices were employed, along with a hip-bath, an aperient, and afterwards a sedative draught. The relief obtained was merely partial and temporary, and the woman died thirty-five hours from the commencement of the attack. In the outward appearance of the stomach, there was nothing to attract notice, but on opening it, its surface was seen to be generally inflamed; and on closely examining it there was found an oval opening in the mucous membrane of the lesser curvature. The submucous coat was also similarly perforated; but the opening in the serous coat was not observable on the outer surface at first sight, but was only recognized (unless very closely inspected,) by passing a probe from the interior of the stomach. It was an oblique slit; and through it about a pint of the stomachic contents had passed into the cavity of the abdomen, causing diffused peritonitis.—Dr. J. B. Thompson, *Med. Gazette*, No. 29. *April* 8, 1842.

---

Two Cases of Glanders in the Human Subject. These two cases, occurring in a man and his wife, followed the usual course, that is, proved utterly unamenable to treatment, and issued fatally. There was no post-mortem examination in the case of the man, and that which was instituted in the woman's case showed merely the lesions usual in such cases. The reporter observes: "In neither of these cases was inoculation accounted for, and we have no reason to believe that the virus was endermically introduced into the system, and indeed the same might be said of many cases on record. I would be much inclined to believe that constantly inhaling an atmosphere saturated with the noxious effluvium from diseased horses would, if incapable of producing the disease, at least very much dispose to the reception of the infection or contagion." The author suggests that the immediate destruction of all glandered horses should be made a business of the public authorities or police.—Dr. DUNNE, *Dublin Med. Press*, No. clxxiv., *May* 4, 1842.

